# Diurnal and Daily Changes in the Levels of Sesquiterpene Lactone and Other Components in Lettuce Post-Harvest

**DOI:** 10.3390/molecules31010080

**Published:** 2025-12-24

**Authors:** Kazuaki Kamata, Hitomi Okada, Yukari Ohta

**Affiliations:** 1 Center for Food Science and Wellness, Gunma University, 4-2 Aramaki, Maebashi 371-8510, Gunma, Japan; kamata.kazuaki@twmu.ac.jp (K.K.); t231a024@gunma-u.ac.jp (H.O.); 2 Institute of Advanced Biomedical Engineering and Science, Tokyo Women’s Medical University, TWIns 8-1 Kawada, Shinjuku-ku, Tokyo 162-8666, Japan; 3 Department of Life and Food Sciences, School of Life and Environmental Sciences, Azabu University, 1-17-71 Fuchinobe, Chuo-ku, Sagamihara 252-5201, Kanagawa, Japan

**Keywords:** lettuce, *Lactuca sativa* L., sesquiterpene lactones, sugar components, H-ORAC values, cichorioside B, lactucin, storage period

## Abstract

Lettuce, which contains sesquiterpene lactones that have been associated with anti-inflammatory and sedative properties, also appears to harbor bitter ingredients such as lactucopicrin, often found abundantly in the emulsion released from the cut core. Previous reports suggest that lettuce may gradually increase in bitterness post-harvest, possibly reflecting alterations in the composition of its components during shelf life. Therefore, analyzing changes in the concentrations of these components could contribute to the development of methods for evaluating lettuce freshness. In this study, we examined variations in sugar contents and hydrophilic oxygen radical absorbance capacity values in lettuce leaves, refined an analytical approach for sesquiterpene lactones in the lettuce core, and explored how their levels may differ depending on harvest timing within the same day and across the storage period. High-resolution LC-MS analysis was employed to estimate the levels of key components such as cichorioside B, 11β,13-dihydrolactucin, lactucin, and lactucopicrin. While the emulsion is generally considered to contain substantial amounts of lactucopicrins, relatively little information is available about the components present in the lettuce core. Our current findings indicate that cichorioside B may be a predominant bitter component in the core. Collectively, these results may provide a basis for developing approaches to assess lettuce freshness and monitor compositional changes during storage.

## 1. Introduction

Lettuce (*Lactuca sativa*) is cultivated worldwide. Four market types are recognized: head, leaf, romaine, and stem. Crisphead lettuce (*Lactuca sativa* L.) is widely consumed in Japan, with Gunma Prefecture being a leading production area. Flavor derives from sugars, organic acids, phenolic compounds, sesquiterpene lactones (SLs) and other related metabolites. Bitterness steadily increases after harvest. This increase is attributed to changes in these compounds during storage.

SLs, a diverse group of terpenoids characterized by lactone rings, are widely distributed in Asteraceae species such as lettuce, chicory, and endive. They play a crucial role in plant defense against abiotic stress, including herbivory [1]. Since the late 20th century, their chemical and sensory properties have been extensively investigated [2,3]. SLs, including lactucin and lactucopicrin, influence bitterness—a sensory trait critical to marketability and nutritional quality—and exhibit pharmacological activities [4,5,6,7]. However, due to the structural diversity of SLs, including novel glycosylated and sulfated derivatives, standardized analytical methods remain insufficiently established [8,9]. Given the global consumption and economic importance of Asteraceae vegetables, the development of reliable analytical approaches for SL-related compounds is essential [5,10].

Free sugars and SLs are primarily responsible for balance of sweetness and bitterness, respectively [11,12]. Recent studies have reported that a higher ratio of sugars to SLs enhances lettuce palatability, as perceived sweetness effectively mitigates SL-derived bitterness [13]. In addition, pre- and post-harvest environmental management—such as light quality and intensity or modified atmosphere packaging—affects physiological processes including respiration, enzymatic activity, and carbohydrate metabolism [14,15,16,17].

Phenolic compounds, such as caffeoylmalic acid and chlorogenic acid, also contribute negatively to taste, although their correlations are weaker than those of SLs [18]. These compounds are also recognized for enhancing antioxidant capacity, a health-related function extensively documented in Asteraceae vegetables, including green and red leaf lettuce [19,20]. Post-harvest polyphenol metabolism is driven by endogenous enzymes that convert phenolics. These activities cause browning, impairing appearance and reducing market value [20,21,22]. Antioxidant capacity, regarded as a biological defense mechanism during the harvest-to-storage transition, is typically assessed using established methods [23,24,25,26]. Changes in antioxidant capacity during storage can serve as indicators of lettuce quality and freshness, together with variations in SLs and free sugars.

Although SLs are critical determinants of lettuce quality, comprehensive analyses of their temporal dynamics in intact cores under refrigerated storage remain scarce. Most previous studies have examined only the white latex exudate from cut surfaces, which differs in composition from the core tissue consumed by consumers. The influence of harvest timing—specifically morning versus evening harvest under varying environmental conditions—on post-harvest component dynamics has not been systematically investigated. Clarifying these factors is essential for developing practical methods of freshness evaluation and for optimizing post-harvest handling.

To address these gaps, we investigated compositional changes in crisphead lettuce during nine days of refrigerated storage. The primary objective was to characterize SLs in lettuce cores using chromatographic separation combined with accurate mass spectrometry. Secondary objectives were to assess harvest timing and to analyze concurrent changes in sugar content and antioxidant capacity in leaf tissue. To reflect consumer and retailer conditions, raw samples were analyzed without freeze-drying, allowing direct evaluation of quality changes in marketable form. These findings provide a basis for chemical marker-based methods to assess lettuce freshness, pending validation through sensory studies.

## 2. Results and Discussion

### 2.1. Lettuce Sample Collection and Processing

**Figure 1:**
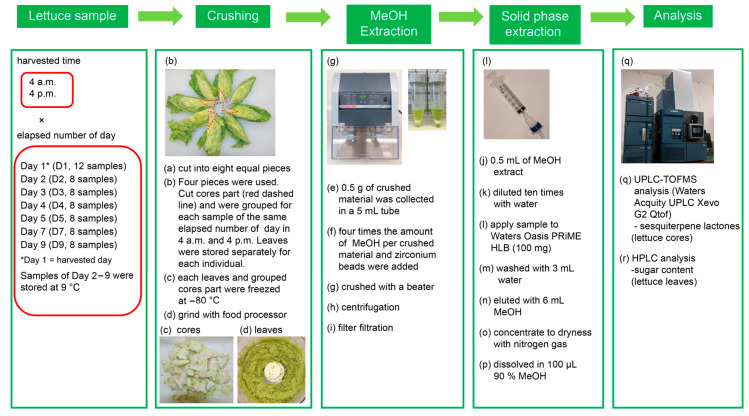
Overview of the testing procedure.

Sample collection and processing with the testing procedure for crisphead lettuce; *Lactuca sativea* L.) are summarized in Figure 1. We compared compositional changes between lettuce harvested at 4 a.m. and 4 p.m. under refrigerated storage (9 °C, up to 9 days). Generally, because lettuce consists of more than 95% water, the freeze-drying process is time-consuming and may introduce changes in the components. In this study, raw samples were deliberately used instead of freeze-dried material to better reflect real-world conditions experienced by consumers and retailers. This approach minimized endogenous enzymatic reactions that may occur even at low temperatures, enabled direct assessment of quality changes in marketable form, and preserved the original tissue structure and water content. At the same time, potential limitations were recognized and controlled: moisture variation was monitored by recording weight changes throughout storage, rapid processing and immediate freezing minimized degradation during handling, and extraction protocols were optimized specifically for raw tissue to ensure reproducibility.

### 2.2. Quantification of Sugars Using High-Performance Liquid Chromatography (HPLC)

**Figure 2:**
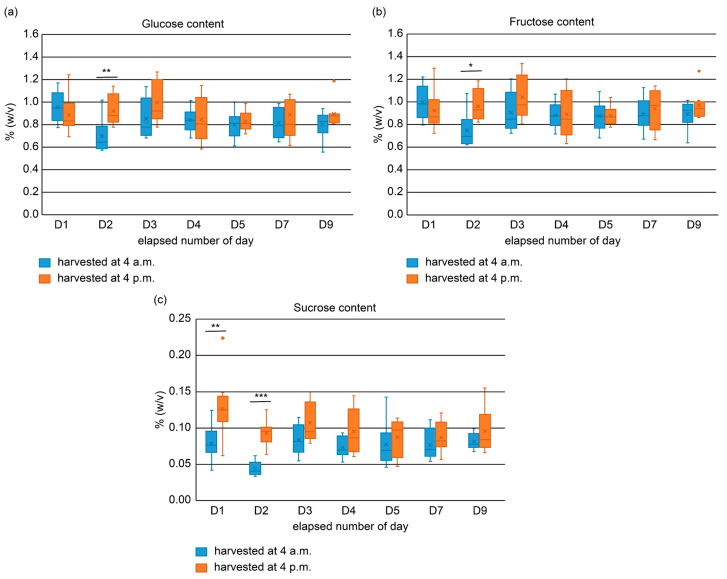
Daily changes in the glucose (**a**), fructose (**b**), and sucrose (**c**) contents of lettuce leaves. The orange dots in the figures for each sugar component are outliers. * *p* < 0.05, ** *p* < 0.01, *** *p* < 0.001.

Glucose, fructose, and sucrose sugars were quantified using HPLC coupled with a refractive index (RI) detector. The glucose contents in specimens harvested at 4 a.m. ranged from 0.5 to 1.2%, whereas those in samples harvested at 4 p.m. ranged from 0.6 to 1.3% (Figure 2). The fructose content ranged from 0.6 to 1.2% at 4 a.m. and 0.6 to 1.3% at 4 p.m. Glucose and fructose contents were significantly different between the samples harvested in the morning and evening of D2. Sucrose levels were 0.03–0.12% for specimens harvested at 4 a.m. and 0.05–0.15% for those harvested at 4 p.m. There was a significant difference in sucrose contents between samples harvested in the morning and evening on D1 and D2, respectively. For the 4 a.m. harvest, the contents of all sugars, including glucose, fructose, and sucrose, declined on D2, followed by an increase on D3. Conversely, specimens harvested at 4 p.m. showed a marginal increase in glucose and fructose contents from D1–D3, followed by a slight decrease on consecutive days. In contrast, the sucrose content decreased slightly from D1 to D2, returned to the D1 level on D3, and then gradually declined. For the sugar content observed on Day 2, circadian regulation of photosynthesis and carbohydrate metabolism is likely to have contributed to the diurnal differences, with daytime accumulation of sucrose and starch and nighttime degradation influencing sugar availability. On Day 3, a recovery in free sugar content was observed. This may indicate the possibility of gluconeogenesis utilizing energy produced by the degradation of storage compounds; however, confirmation of this hypothesis would require a broader analysis of storage compounds and their metabolites. This interpretation is supported by studies showing circadian regulation of carbohydrate metabolism in plants [14].

### 2.3. Antioxidant Capacity

**Figure 3:**
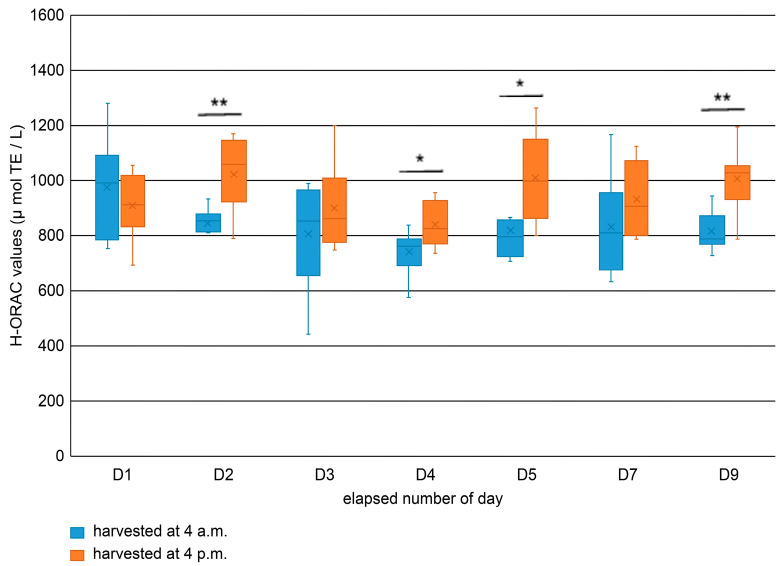
Daily changes in hydrophilic-oxygen radical absorbance capacity (H-ORAC) values of lettuce leaves. * *p* < 0.05, ** *p* < 0.01.

Hydrophilic oxygen radical absorbance capacity (H-ORAC) measurement results suggested that the harvest at 4 a.m. exhibited overall values ranging from 400 to 1300 μmol TE/L, whereas those harvested at 4 p.m. ranged between 700 and 1300 μmol trolox equivalents (TE)/L, with the lowest values being higher for those harvested at 4 p.m. (Figure 3). H-ORAC values showed significant differences between samples harvested in the morning and evening on D2, D4, D5, and D9. The 4 a.m.-harvested specimens showed a moderate decline in H-ORAC values from D1, whereas the values of those harvested at 4 p.m. declined until D4 but increased again at D5. The tendency for the H-ORAC values to be higher in samples harvested in the evening was presumably due to the higher temperature at harvest time, which resulted in the more rapid synthesis of polyphenols with antioxidant activity, compared to that in samples harvested in the morning. Fluctuations in antioxidant capacity observed during storage are likely influenced by changes in enzymatic activities, such as those of polyphenol oxidase and phenylalanine ammonia-lyase. The increase observed in the 4 p.m. samples could be linked to stress-induced polyphenol biosynthesis, serving as a protective mechanism activated by harvest stress and exposure to sunlight at higher temperatures during the transition from field to storage. These findings highlight the dynamic nature of antioxidant metabolism in lettuce post-harvest.

### 2.4. Identification and Analysis of Sesquiterpene Lactones

**Figure 4:**
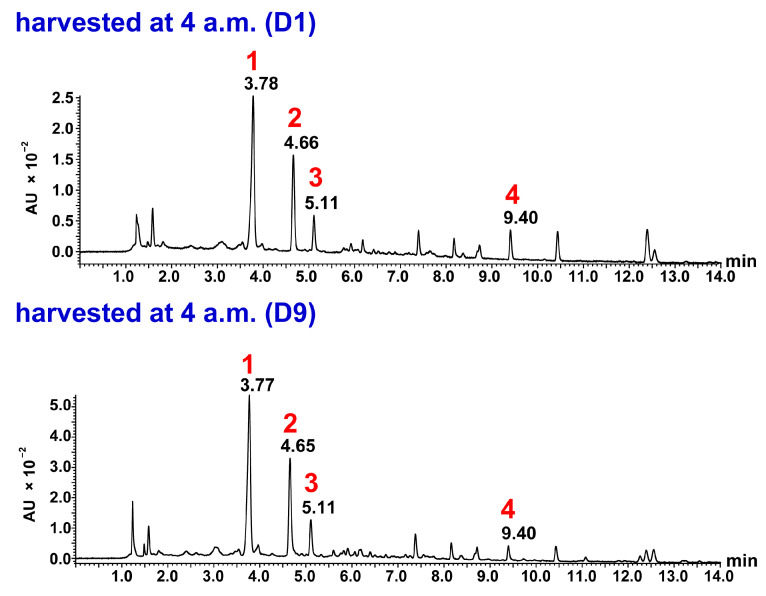
UPLC chromatograms of lettuce cores harvested at 4 a.m. on D1 and D9 (UV 262 nm). Conditions: Waters ACQUITY UPLC BEH C18 column (1.7 μm and 2.1 × 100 mm). The mobile phase consisted of Liquid A (water with 10 mM ammonium acetate) and Liquid B (MeCN with 10 mM ammonium acetate), with a Liquid A/Liquid B ratio transitioning from 90:10 to 40:60 over 0–15 min, then to 30:70 from 15.1 to 16 min, and finally returning to 90:10 from 16.1 to 25 min; column oven temperature: 35 °C; Detector: UV 262 nm; flow rate: 0.2 mL/min; injection volume: 3 μL.

**Figure 5:**
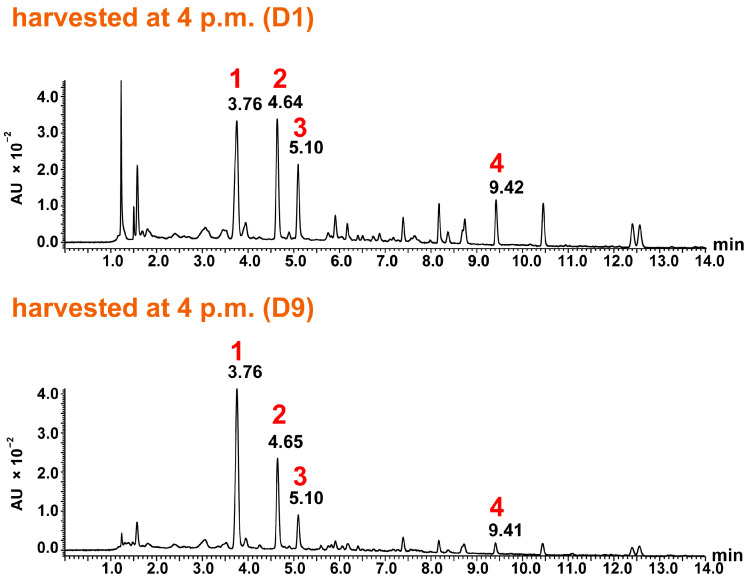
UPLC chromatograms of lettuce cores harvested at 4 p.m. on D1 and D9 (UV 262 nm). The analytical conditions were the same as in Figure 4.

**Figure 6:**
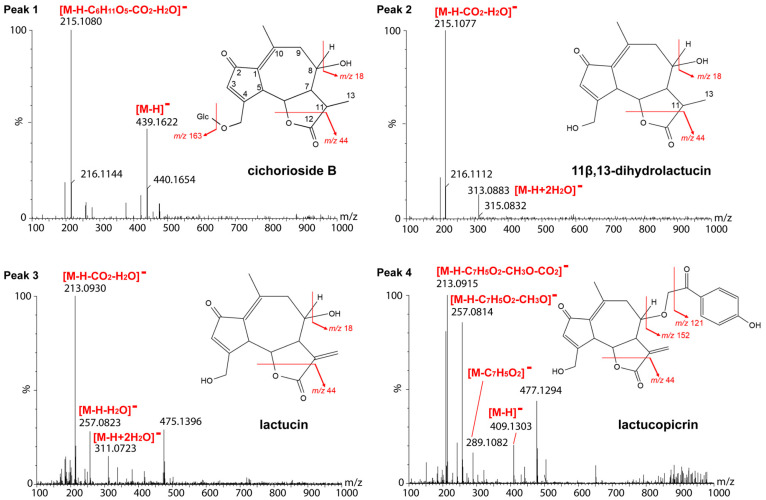
Mass spectra of each peak in the UPLC chromatogram and chemical structure of estimated sesquiterpene lactones. For each compound, the fragmentation pattern is shown. Mass spectra were acquired in negative ion mode over the *m/z* range of 100–1000. The detected MS signals were assigned based on the predicted fragmentation patterns derived from the structures and molecular formulas of the putative SLs. Details of these assignments are provided in Section 2.4. of the main text.

Ultra-performance liquid chromatography–time of flight-mass spectrometry (UPLC-TOF-MS) was performed in negative ion mode as sesquiterpene lactones—particularly the carboxylated and glycosylated derivatives such as cichorioside B and 11β,13-dihydrolactucin derivatives—show superior ionization efficiency in negative mode due to their acidic functional groups. Previous studies have demonstrated that negative ion mode provides better sensitivity and more stable signals for these compounds [8,9]. Most of the signals predicted to be parent ions, adduct ions, and fragment ions were observed at -H. And it revealed that peaks 1–4 (Figure 4 and Figure 5) had a maximum absorption around 262 nm and similar UV spectra as detected by a photodiode array detector, suggesting that they were related compounds. Based on our UPLC-TOF-MS analysis and the results from the literature [8], peak 1 was identified as cichorioside B (observed: *m*/*z* 439.1622, C_21_H_27_O_10_, calculated: *m*/*z* 439.1604, +1.8 mDa). In peak 1, a signal was observed at *m*/*z* 215.1080, which was presumably due to glucose elimination, dehydration, and decarboxylation after lactone cleavage. In the MS spectrum of peak 2, a signal at *m/z* 313 was observed, which was thought to be due to the addition of two water molecules, and in addition, the observed signal at *m*/*z* 215.1077 was presumably due to the cleavage of lactone, followed by decarboxylation and dehydration, as with peak 1. This process suggested that peak 2 likely represented 11β,13-dihydrolactucin (calculated: *m/z* 215.1072, C_14_H_15_O_2_, +0.5 mDa). Chromatograms with UPLC showed that peak 3 had the same retention time as a lactucin standard, and therefore, it was presumed to be lactucin. Moreover, in peak 3, a signal was observed at *m*/*z* 311, which was assumed to be due to the addition of two water molecules, and a signal was observed at *m*/*z* 257.0823, which was due to the dehydration of one water molecule. In addition, a signal was observed at *m*/*z* 213.0930, which was due to dehydration and lactone cleavage followed by decarboxylation. Therefore, peak 3 was identified as lactucin (calculated: *m/z* 257.0814, C_15_H_13_O_4_, −1.0 mDa). Peak 4 was presumed to be lactucopicrin because its retention time matched that of a lactucopicrin standard in the UPLC chromatograms. Peak 4 showed a [M-H]^−^ signal at *m*/*z* 409.1303, and fragment ion peaks at *m*/*z* 289.1082 and *m*/*z* 257.0814 suggested the loss of benzoyl and hydroxymethyl. A signal at *m*/*z* 213.0915 suggested that the compound was decarboxylated, similar to the compound in peak 3. Thus, peak 4 was identified as lactucopicrin (calculated: *m*/*z* 409.1287, C_23_H_21_O_7_, +1.6 mDa). In the MS spectra of lactucin and lactucopicrin, signals at *m*/*z* 213 were observed, indicating the exomethylene type at positions 11 and 13. Cichorioside B and 11β,13-dihydrolactucin signals were observed at *m*/*z* 215, suggesting that positions 11 and 13 were the dihydro form (Figure 6). Cichorioside B was identified as a major component in the lettuce core, and changes in the amounts of these components over the storage period were investigated.

Lactucopicrin has been reported as a major bitter compound in lettuce, particularly abundant in the white latex emulsion released from specialized laticiferous tissues, known as latex canals, which are part of the plant’s vascular system. Arakawa et al. reported that the core of crisphead lettuce contains lactucopicrin and 8-deoxy lactucin and investigated their contents at each growth stage [12]. In this study, we newly discovered that the core contains chicorioside B and 11β,13-dihydrolactucin. Our analysis conducted on the whole core parts, which shows a different composition. The predominance of cichorioside B in the core suggests tissue-specific distribution of sesquiterpene lactones, with different compounds accumulating in different parts of the plant. This finding is important because consumers typically consume the core tissue rather than only the latex emulsion, making cichorioside B a more relevant marker for assessing quality of edible portions.

### 2.5. Quantitative Analysis of Sesquiterpene Lactones and Evaluation of Freshness

**Figure 7:**
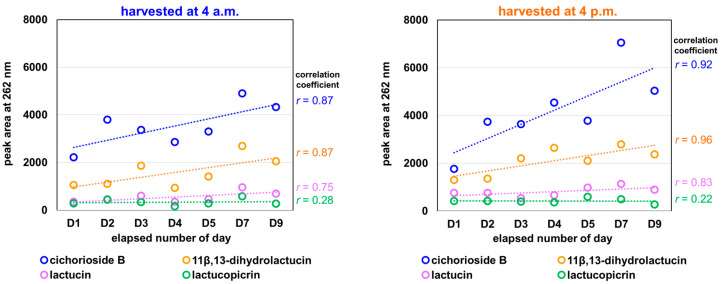
Diurnal changes in each sesquiterpene lactone compound in the core of lettuce harvested at 4 a.m. and 4 p.m. The peak area values at UV262 nm for each sesquiterpene lactone at each elapsed day were plotted and expressed as a regression line with correlation coefficient (*r*). Changes in the peak areas of sesquiterpene lactones during storage. Peak areas at 262 nm are shown as semi-quantitative measures.

The changes in each component in lettuce harvested at 4 a.m. and 4 p.m. during each storage period are summarized in Figure 7, and each is represented as a regression line.

Major components like cichorioside B and 11β,13-dihydrolactucin exhibit linearity (r > 0.92) in the samples at 4 p.m., whereas those at 4 a.m. showed moderate correlation (r = 0.87). The 4 p.m. sample sets accumulated compounds more rapidly. Notably, cichorioside B increases at nearly double the rate in the 4 p.m. samples (slope: 437) compared to the 4.0 a.m. samples (slope: 260). In both sample sets, lactucopicrin is the only component that shows no linear correlation, indicating that its levels fluctuate independently from time. Collectively, these findings suggest that the cichorioside B content in the core of lettuce may serve as an indicator of its freshness.

## 3. Materials and Methods

### 3.1. Chemicals and Reagents

For the analysis and sample preparation, LC/MS-grade methanol, acetonitrile, and ultrapure water, obtained from FUJIFILM Wako Pure Chemical Co., Ltd. (Osaka, Japan), were used. HPLC-grade 1 M ammonium acetate and special-grade methanol were also used. Lactucopicrin and lactucin were procured from Funakoshi Co., Ltd. (Tokyo, Japan). Ethyl ferulate was sourced from Tokyo Chemical Industries, Ltd. (Tokyo, Japan). D-Glucose and sucrose were acquired from Nacalai tesque, Inc. (Kyoto, Japan), and D-fructose was obtained from FUJIFILM Wako Pure Chemical Co., Ltd.

### 3.2. Plant Specimens

In this study, crisphead lettuce (*Lactuca sativa* L.) cultivar oasis was used. They were sown on 4 April 2022, planted on 27 April, and harvested on 16 June, grown in the same field in Numata City, Gunma Prefecture (36 degrees 40 min 5.8764 s north, 139 degrees 1 min 24.582 s east). Sixty lettuce samples were harvested in the morning (at 4 a.m.), and 60 additional samples were harvested in the evening (at 4 p.m.). Specimen storage and processing methods for lettuce were described previously herein.

This study was resource-constrained, and the sample size for this experiment was chosen as the maximum practically possible based on the physical constraints of the laboratory, including sample preparation, instrumentation and reagent costs, and the time and manpower available for analysis.

### 3.3. Extraction of Plant Material

Sixty Crisphead lettuce (*Lactuca sativa* L.) samples cultivated in the same field were harvested at 4 a.m. (temperature: 13 °C, humidity: 100%), and 60 additional samples were harvested at 4 p.m. (temperature: 22 °C, humidity: 75%). On the harvesting day, one or two outer leaves of 120 lettuce samples were peeled off, and the weight of each sample was measured. After measuring the weight, the cut surfaces of the samples were covered with a Kimtowel, and each sample was placed in a plastic bag. For sample retrieval on the harvest day, this was considered the first day, and subsequently, refrigerated samples at 9 °C were periodically retrieved. The lettuce harvest day was denoted as the first day (D1), and days 2, 3, 4, 5, 7, and 9 were labeled as D2, D3, D4, D5, D7, and D9, respectively. Twelve samples harvested at 4 a.m. were cut into eight equal parts with a ceramic knife at 7 a.m., whereas 12 samples harvested at 4 p.m. were cut at 7 p.m. The core, denoted by the red dotted line in Figure 1, was then cut. The cut leaves were individually placed in plastic bags and frozen at −80 °C on D1. The cut cores were grouped into the 4 a.m. and 4 p.m. groups, placed in plastic bags, and frozen at −80 °C. The remaining 96 lettuce samples were stored at 9 °C in a chamber, with eight samples harvested at 4 a.m. and eight samples harvested at 4 p.m. being processed on D2 in a similar manner to the process used for D1. This process was repeated on days 3, 4, 5, 7, and 9, and the resulting samples were denoted as D3, D4, D5, D7, and D9. The frozen lettuce leaf portion was crushed in a food processor, and 0.5 g of the crushed material was collected in a 5 mL tube (Eppendorf, Hamburg, Germany). Four times the amount of methanol and zirconium beads was added, and the material was crushed with a Beads crusher μT-12 (TAITEC, Saitama, Japan) for 2 min at 2500 r/min to extract the desired components from the plant material. The frozen core portion was subjected to a similar crushing process, and methanol extraction was performed using a beater.

### 3.4. Sugar Analysis

The methanol extract of the lettuce was centrifuged (14000× *g*, 4 °C, 15 min), and the supernatant was filtered using an Amicon^®^ Ultra Centrifugal Filter, 10 kDa molecular weight cut-off, 0.5 mL sample volume (Merck, Germany), which helped to retain components with a molecular weight >10 kDa. The resulting filtrate was used for sucrose analysis after being diluted twice with acetonitrile, as well as for glucose and fructose analysis. HPLC (Alliance e2695, Waters Corporation, Milford, MA, USA) was performed using a high-performance carbohydrate column (φ 4.6 × 250 mm, 60 Å, 4 μm, Waters) coupled with a 2414 RI detector (Waters Corporation, Milford, MA, USA). The flow rate was set at 1.0 mL/min, the column oven temperature was maintained at 50 °C, and the sample was injected and analyzed. A mobile acetonitrile:water phase of 85:15 was used for the analysis of D-glucose and D-fructose, whereas that of 80:20 was employed for the analysis of sucrose. The calibration curves were drawn using the standards of glucose, fructose and sucrose, and the content of each component was calculated. Paired comparisons were analyzed using Student’s *t*-tests. One, two, and three asterisks indicate *p* < 0.05, *p* < 0.01, and *p* < 0.001, respectively. The Levene test showed *p* > 0.05 for all days, indicating that homogeneity of variance was appropriate, and the Shapiro–Wilk test showed *p* > 0.05 for all groups, indicating that normality was appropriate. Therefore, the results of this *t*-test were deemed to be valid.

### 3.5. H-ORAC Analysis

The methanol extract of the lettuce leaves was centrifuged (14000× *g*, 4 °C, 15 min), and then, the supernatant was filtered using the 0.22 μm filter. This MeOH extract was used as measure samples. A kit for H-ORAC measurement was procured from FUJIFILM Wako Pure Chemical Co., Ltd., and samples were prepared and measured using a fluorescence plate reader (EnSpire Multimode Plate Reader, PerkinElmer, Waltham, MA, USA) according to the manufacturer’s instructions. Paired comparisons were analyzed using Student’s *t*-test. One and two asterisks indicate * *p* < 0.05 and ** *p* < 0.01, respectively.

### 3.6. UPLC-TOF-MS Analysis

The methanol extracts of the lettuce core portion harvested in the morning and evening (D1, D2, D3, D4, D5, D7, and D9) were centrifuged (14000× *g*, 4 °C, 30 min), and then the supernatant was filtered using the 0.22 μm filter. Next, 500 μL of the filtrate was taken, 100 μL of the 0.05 mM ethyl ferulate (Rt 12.3–12.6 min), as an internal standard, was added to each filtrate, and then each solution was diluted 10 times with ultrapure water. Solid-phase extraction using Oasis PRiME HLB 100 mg (Waters) was performed, and the methanol eluate was concentrated and dried with N_2_ gas. Subsequently, 100 μL of 90% methanol was added, and the solution was dissolved to obtain an analyte. The UPLC-TOF-MS analysis was performed on a Waters ACQUITY UPLC BEH C18 column with dimensions of 1.7 μm and 2.1 × 100 mm at 35 °C. (The inlet system was Waters Acquity H-class UPLC system, and the MS system was a Waters Xevo G2 quadrupole time-of-flight mass spectrometer). The mobile phase consisted of Liquid A (water with 10 mM ammonium acetate) and Liquid B (MeCN with 10 mM ammonium acetate), with a Liquid A/Liquid B ratio transitioning from 90:10 to 40:60 over 0–15 min, then to 30:70 from 15.1 to 16 min, and finally returning to 90:10 from 16.1 to 25 min. The flow rate was maintained at 0.2 mL/min, and an injection volume of 3 μL was utilized. Mass range 100–1000 Da, desolvation temperature 500 °C, source temperature 120 °C, scan time 0.5 s. A photodiode array (PDA) detector was used for monitoring the absorbance at 262 nm. The peak area for each substance in peaks 1–4 was corrected based on the recovery rate of ethyl ferulate, which was used as an internal standard. As the UV spectra and maximum absorption were almost identical for the four identified components, the quantification was conducted by comparing with the peak area value at UV 262 nm.

Statistical Analysis was conducted to evaluate the relationship between the sample progression (storage duration) and the content of each sesquiterpene lactone, and simple linear regression analysis was performed. The strength of the linear association was assessed using Pearson’s product-moment correlation coefficient (*r*). The slope of the regression line was calculated to estimate the accumulation rate of each compound.

For sesquiterpene lactones lacking authentic standards (cichorioside B and 11β,13-dihydrolactucin), peak areas at 262 nm were used as semi-quantitative measures to compare relative changes during storage.

## 4. Limitations of This Study

An important limitation of the current study is the absence of direct sensory evaluation data. While our chemical analyses demonstrated significant increases in cichorioside B and other sesquiterpene lactones during storage, we cannot definitively conclude that these chemical changes directly correlate with perceived bitterness or a decline in freshness without sensory validation. Although previous studies have established correlations between sesquiterpene lactone concentrations and bitterness perception [5,7], the relationship between chemical composition and human sensory perception is complex. Factors such as the food matrix effect, interactions between bitter and sweet compounds (e.g., the balance between sugars and sesquiterpene lactones), and individual variations in taste sensitivity can all influence perceived quality. Therefore, while the chemical markers identified in this study provide a valuable scientific foundation, future research incorporating trained sensory panels is essential to validate their practical utility as predictors of consumer acceptability and freshness.

## 5. Conclusions

This study elucidated the temporal changes in sugar contents, H-ORAC values, and sesquiterpene lactones in lettuce from harvest through storage. While sugar components exhibited transient fluctuations, harvest timing proved influential for antioxidant capacity, with evening harvests maintaining higher H-ORAC values likely due to temperature-induced biosynthesis. Regarding bitter components, UPLC-TOF-MS analysis identified cichorioside B as the predominant constituent in the lettuce core. Notably, our approach using raw samples provided distinct insights into the native composition of fresh lettuce compared to conventional lyophilized samples. These chemical characteristics may provide a foundation for developing objective freshness evaluation methods.

From a practical perspective, these findings offer valuable insights for the agricultural supply chain. For growers, optimizing harvest timing based on these metabolic rhythms could help maximize sugar content and manage initial bitterness levels. For distributors and retailers, understanding the stability of these components allows for optimized storage conditions and potentially enables quality monitoring based on chemical markers, leading to improved inventory management and objective quality assessment.

However, the chemical markers identified in this study have the potential for use as freshness indicators rather than definitive measures at this stage. Validation studies incorporating sensory evaluation, consumer acceptability tests, and assessment across different cultivars and growing conditions will be necessary before the practical implementation of this approach. Future research should expand on these findings to bridge the gap between chemical composition and consumer-perceived quality.

## Data Availability

Data is contained within the article.

## Abbreviations

UPLC-TOF-MS	Ultra-performance liquid chromatography–time of flight-mass spectrometry
H-ORAC	Hydrophilic oxygen radical absorbance capacity
HPLC	High-performance liquid chromatography
PDA	Photodiode array
